# A Novel Signaling Driven by the Stem Cell Marker ALDH1A3 Promotes Glioblastoma Cell Mobility

**DOI:** 10.3390/cells15121079

**Published:** 2026-06-14

**Authors:** Zhong-Rong Chen, Zhen Chen, Qiang Dong, Rainer Will, Maike Anna Busch, Nicole Dünker, Philipp Dammann, Ulrich Sure, Yuan Zhu

**Affiliations:** 1 Department of Neurosurgery and Spine Surgery, University Hospital Essen, University of Duisburg-Essen, 45147 Essen, Germany; zhong-rong.chen@uk-essen.de (Z.-R.C.); zhen.chen-neurosurgery@outlook.com (Z.C.); dongq19@lzu.edu.cn (Q.D.); philipp.dammann@uk-essen.de (P.D.); ulrich.sure@uk-essen.de (U.S.); 2 Center for Translational Neuro- and Behavioral Sciences (C-TNBS), University Hospital Essen, University of Duisburg-Essen, 45147 Essen, Germany; maike.busch@uk-essen.de (M.A.B.); nicole.duenker@uk-essen.de (N.D.); 3 Core Facility Cellular Tools, German Cancer Research Center (DKFZ), 69120 Heidelberg, Germany; r.will@dkfz-heidelberg.de; 4 Department of Neuroanatomy, Medical Faculty, Institute of Anatomy II, University of Duisburg-Essen, 45147 Essen, Germany

**Keywords:** glioblastoma, ALDH1A3, retinoic acid and retinoic acid receptors, PAI-1, invasion, migration, CRISPR/Cas9

## Abstract

Glioblastoma (GBM) is an extremely invasive and incurable tumor. We previously reported predominant ALDH1A3 expression at the invasive front of GBM tumors, which was associated with shorter patient survival, and further showed that ALDH1A3 promoted tumor angiogenesis involving plasminogen activator inhibitor-1 (PAI-1). Here, we investigated whether ALDH1A3 drives cell invasion through retinoic acid (RA) and PAI-1 signaling. Analysis of the TCGA-GBM dataset revealed a positive association between ALDH1A3 and PAI-1 (SERPINE1) expression. Overexpression of ALDH1A3 in GBM cells markedly increased PAI-1 mRNA and protein levels, with cellular colocalization of both proteins, accompanied by robust migration and invasion. These effects were reversed by treatment with a pan-RA receptor (RAR) antagonist AGN193109 (AGN), with a specific PAI-1 inhibitor tiplaxtinin (Tip) or by CRISPR/Cas9-mediated knockout of PAI-1. In a chick chorioallantoic membrane (CAM) model, ALDH1A3-overexpressing cells showed increased invasion, which was reduced by tiplaxtinin (Tip) treatment or PAI-1 knockout. Mechanistically, ChIP-qPCR demonstrated that RA treatment or ALDH1A3 overexpression increased RARα occupancy at the PAI-1 regulatory region, accompanied by increased PAI-1 expression, both of which were diminished by AGN. Collectively, the present study defines an ALDH1A3-RA-PAI-1 signaling axis that contributes to GBM cell motility and invasion.

## 1. Introduction

Glioblastoma (GBM) is an extremely aggressive and incurable malignancy characterized by diffusely infiltrative growth. Its invasive nature leads to a high recurrence rate and short survival, typically less than two years, making it extremely challenging to treat [1]. Targeting the migratory and invasive phenotype of GBM may reduce the spread of residual tumor cells after surgical resection, increase survival, and facilitate surgical re-resection in the recurrence setting. However, the biology of malignant cells at the GBM invasive front, despite its significance, has not been elucidated in detail.

The aldehyde dehydrogenase (ALDH) family is a group of enzymes that consists of 19 isoforms in humans and are localized in the cytoplasm, nucleus or mitochondria. The main biological functions of ALDHs include the irreversible metabolism of endogenous and exogenous aldehydes to carboxylic acids and reducing lipid peroxidation as a reactive oxygen species (ROS) scavenger thereby attenuating oxidative stress. ALDH1A3 is a member of the ALDH family and shows higher enzyme activity in GBM than other family members [2]. It serves as a stem cell marker and is a key driver of proneural-to-mesenchymal transition (PMT); accordingly, it is widely recognized as a marker of glioma stem cells and mesenchymal GBM, the most aggressive type of GBM [3,4]. In cell-based functional assays, downregulation of ALDH1A3 diminished cell proliferation, invasion, glycolysis and self-renewal capacity, and reduced expression of mesenchymal markers [2,5,6]. Xenograft models have demonstrated that high ALDH1A3 activity is involved in tumor initiation, invasiveness, angiogenesis, glycolysis, and therapy resistance [2,5]. In human GBM, ALDH1A3 expression is associated with aggressive biology and poor clinical outcome as shown by reduced responses to ionizing radiation [6], and to postoperative chemoradiotherapy in ALDH1A3-high GBM [7]. Although ALDH1A3 has been linked to several aggressive GBM phenotypes, it remains unclear how it is connected to the regulation of GBM cell migration and invasion.

Plasminogen activator inhibitor-1 (PAI-1) is a protein long implicated in GBM invasion. Multiple studies have shown that diverse upstream cues, including SMAD2/3 signaling, regulate PAI-1 to trigger cell migration and invasion [8]. Genetic or pharmacological inhibition of PAI-1 reduces GBM cell invasiveness in orthotopic models [9]. However, it remains unknown whether it serves as a downstream mediator linking ALDH1A3-related RA signaling to GBM cell mobility.

We have reported that ALDH1A3 resides predominantly at the invasive front of GBM and was negatively associated with patients’ overall survival time, highlighting its potential prognostic and clinical relevance [10]. Moreover, we have recently shown that ALDH1A3 promotes tumor angiogenesis through multiple proangiogenic factors, including PAI-1 [11]. Given the major biological function of ALDH1A3 in catalyzing the irreversible oxidation of retinaldehyde to retinoic acid (RA) [12], it is therefore of great interest to explore whether ALDH1A3-associated RA signaling is associated with PAI-1-dependent GBM cell mobility and, if so, how ALDH1A3 regulates PAI-1 through RA/RAR signaling in GBM cells. We hypothesized that ALDH1A3 may drive a signaling axis involving RA and PAI-1 in the activation of GBM cell migration and invasion. To this end, the present study uses genetically engineered GBM cells in various in vitro and in vivo models to address the role of the ALDH1A3-RA/RAR-PAI-1 signaling in GBM cell mobility. The present study provides both functional evidence and a mechanistic framework for understanding the crucial role of ALDH1A3 in GBM cell migration and invasion.

## 2. Materials and Methods

### 2.1. Cell Culture and Generation of Ev/oxALDH1A3 GBM Cell Lines

U373 and LN229 cells were maintained in a growth medium consisting of Dulbecco’s Modified Eagle Medium (DMEM, cat# 61965026, Gibco, Waltham, MA, USA), 10% fetal bovine serum (FBS) and 1% sodium pyruvate at 37 °C in a humidified incubator with 5% CO_2_. ALDH1A3-overexpressing (ox) and empty-vector (ev) GBM cell lines were generated by lentiviral transduction as previously described [11]. These transduced cells were cultured in the growth medium supplemented with blasticidin (5 μg/mL; cat# A1113902; Thermo Fisher, Waltham, MA, USA) as selection medium.

### 2.2. CRISPR/Cas9-Mediated PAI-1 Knockout

The PAI-1 gene was knocked out in ALDH1A3-overexpressing (ox) and empty-vector (ev) U373 cells by CRISPR/Cas9-mediated genome editing. Briefly, ribonucleoprotein (RNP) complexes were assembled by incubating 180 pmol predesigned synthetic guide RNA CRISPRevolution sgRNA SERPINE1-101128416 GCCCAGGACUAGGCAGGUGA (Synthego; Redwood City, CA, USA) targeting human PAI-1 with 20 µM TrueCut™ Cas9 Protein v2 (Thermo Fisher). For nucleofection of the RNP complex, 3 × 10^5^ cells (ev-/oxU373) per reaction were pelleted by centrifugation and resuspended in 20 µL SF Cell Line 4D-Nucleofector buffer (Lonza, Basel, Switzerland). Cell suspension and RNP complexes were mixed and nucleofected in 16-well strips using program DS-138 on a 4D-Nucleofector device (Lonza). Following nucleofection, cells were transferred into two 12-well plates and cultured until approximately 60% confluency. One well was used for genotyping of the edited pool, while the second well was expanded for subsequent single-cell cloning.

Knockout efficiency was assessed by genomic DNA extraction using QuickExtract DNA Extraction Solution (Biozym, Hessisch Oldendorf, Germany), PCR amplification of the targeted locus (PAI_F: AAGATTCCCACAGGGCAAGA; PAI_R: CTGCTTGAATCTGCTGCTGG), followed by Sanger sequencing (PAI_Seq: ACCTGCTTGCAGGAAACAAG; Eurofins Genomics, Ebersberg, Germany) and ICE analysis (Inference of CRISPR Edits) [13]. Potential off-target sites for the selected guide RNA were assessed in silico using CRISPOR against the human hg38 reference genome. The guide RNA showed an off-target profile of 0-0-2-31-284, with no predicted off-target sites containing 0 or 1 mismatch. The two predicted 2-mismatch candidate sites were located in intronic regions and had low CFD scores of 0.17 and 0.12, respectively, indicating a low predicted risk of coding off-target cleavage. Pools with knockout efficiencies greater than 90% were expanded and subjected to single-cell dispensing into 96-well plates using the F.sight single-cell dispenser (Cytena, Freiburg, Germany). Single-cell clones were expanded and genotyped as described above and the PAI-1 k.o. was further validated by Western blotting.

### 2.3. Lentiviral GFP Transfection in GBM Cell Lines

HEK293T cells were transfected with 6 µg of GFP-expressing plasmid (pCL7EGwo; kindly provided by Dr. H. Hanenberg) using polyethyleneimine (PEI, branched; Sigma-Aldrich, St. Louis, MO, USA; 45 µg) as previously described [14]. The medium was replaced 24 h after transfection with IMDM (Gibco, Waltham, MA, USA) supplemented with 10% FCS and 1% penicillin/streptomycin. Viral supernatants were collected 48 h post-transfection, filtered (0.45 µm), and cryopreserved. For stable GFP labeling, GBM cells were seeded in 6-well plates (1 × 10^5^ cells/well) and incubated for 24 h with GFP-lentiviral supernatant in the presence of polybrene (10 µg/mL; H9268; Sigma-Aldrich, St. Louis, MO, USA). After 24 h, the medium was replaced with 2 mL selection medium. Cells were subcultured for expansion after reaching 70% confluence.

### 2.4. TCGA Dataset Analysis

Transcriptomic and clinical data for the TCGA-GBM cohort were obtained from the GlioVis data portal [15]. For correlation analysis, samples annotated as glioblastoma (GBM) in the histology category of the GlioVis TCGA_GBM RNA-seq platform were downloaded (*n* = 153). Cases annotated as IDH-mutant or without available IDH annotation were excluded (*n* = 11), and the remaining IDH-wildtype GBM (*n* = 142) cases were used for correlation analysis. Correlations between ALDH1A3 and SERPINE1/PAI-1 expression were assessed in RStudio (v2026.1.1.403) using Spearman’s rank correlation analysis. For survival analyses, the TCGA-GBM HG-U133A platform available in GlioVis was additionally used, and analyses were performed for all included cases and for the IDH-wildtype subgroup where annotation was available. Patients were stratified according to ALDH1A3 or SERPINE1/PAI-1 expression. High- and low-expression groups were defined using the lower-quartile expression cut-off implemented in GlioVis, i.e., the low-expression group included patients within the lowest 25% of expression values, whereas the high-expression group included the remaining 75% of patients above this lower-quartile cut-off. Kaplan–Meier survival curves were compared between the high- and low-expression groups using the log-rank test, and hazard ratios (HRs) with 95% confidence intervals (CIs) were obtained using Cox proportional hazards regression, as implemented in GlioVis.

### 2.5. Double Immunofluorescence (IF) Staining

Double IF staining was performed as previously described [16]. The following antibody mixtures were applied: rabbit anti-ALDH1A3 (1:250; cat# NBP2-15339; Novus Biologicals, Centennial, CO, USA) combined with mouse anti-PAI-1 (1:200; cat#: 66261-1-Ig; Proteintech, Wuhan, China). Cell nuclei were counterstained with DAPI (Thermo Fisher Scientific, Schwerte, Germany). Images were acquired using an AxioImager M.2(Carl Zeiss AG, Oberkochen, Germany) microscope under identical acquisition settings across groups.

### 2.6. Western Blotting

Protein extraction, electrophoresis, and immunoblotting were performed as previously described [11]. Membranes were blocked with 5% non-fat milk and incubated overnight at 4 °C with primary antibodies: ALDH1A3 (1:1000; cat# NBP2-15339; Novus Biologicals, Centennial, CO, USA), PAI-1 (1:1000; cat# 66261-1-Ig; Proteintech, Wuhan, China) and GAPDH (1:2000; cat# 2118; Cell Signaling Technology, Danvers, MA, USA). For semi-quantitative analysis, the integrated optical density (IOD) of immunoreactive bands was measured using ImageJ software (v1.1.53t). Protein expression was normalized to GAPDH, and values were presented as a percentage relative to the control.

### 2.7. Scratch Assay

Cell migration was assessed using a scratch assay as previously described [16]. Briefly, 5 × 10^5^ GBM cells were seeded in a 6-well plate and incubated overnight. A uniform scratch was introduced using a sterile 100 µL pipette tip, and wound closure was monitored at 12 h and 24 h under a microscope (5× objective). The wound healing area was quantified as the percentage of the uncovered scratched area compared to 0 h using ImageJ.

### 2.8. Transwell Migration and Invasion Assay

Cell migration and invasion were measured using 24-well plates with inserts (8 μm pore) (cat# 3422; Corning, NY, USA). For the migration assay, inserts were used uncoated. For the invasion assay, inserts were pre-coated with Matrigel (0.5 mg/mL; cat# 356234; Corning) and allowed to solidify at 37 °C for 1 h (coating volume: 100 μL/insert).

For each assay, 5 × 10^4^ cells suspended in 200 μL DMEM were seeded into the upper chamber. The lower chamber was filled with 700 μL complete medium (DMEM + 10% FBS) as a chemoattractant, with or without pharmacological inhibitors as indicated. Cells were incubated for 12 h (migration) or 24 h (invasion) at 37 °C. Following incubation, non-migrated or non-invaded cells were removed using a cotton swab, and cells on the underside were fixed with 4% paraformaldehyde and stained with 0.5% crystal violet. Images were acquired under a 20× objective, and cells were quantified by counting five randomly selected fields per insert.

### 2.9. Spheroid Invasion Assay

Three-dimensional cell invasion was assessed using a spheroid-based invasion assay, with modifications as previously described [11]. GBM cells were suspended in 25 μL selection medium containing 20% methylcellulose and seeded into a 96-well U-bottom plate. After incubation overnight, formed spheroids were embedded in 50 μL Matrigel-containing medium (40% Matrigel) and incubated at 37 °C for 60 min. Subsequently, 50 μL fresh selection medium with or without inhibitors was added per well. Invasion was monitored at 48 h and 72 h post-embedding. Images of six spheroids per group were acquired using a microscope equipped with a 5× objective, and the invaded area was quantified using ImageJ.

### 2.10. In Silico Motif Analysis

The PAI-1 regulatory region (±2 kb of the transcription start site, hg38) was analyzed by in silico motif prediction using the JASPAR web interface. Predicted RAR/RXR binding motifs were identified by scanning JASPAR position weight matrices (PWMs) for RARα: RXR heterodimers, and the top-scoring sites were selected for downstream analyses. Motif locations and scores were exported from the JASPAR output for figure preparation.

### 2.11. Chromatin Immunoprecipitation-Quantitative PCR (ChIP-qPCR)

ChIP assays were performed using the SimpleChIP Enzymatic Chromatin IP Kit (cat# 9002; Cell Signaling Technology, Danvers, MA, USA) according to the manufacturer’s instructions. Cells were cross-linked with 1% formaldehyde for 10 min at room temperature and quenched with 125 mM glycine. Nuclei were isolated and chromatin was digested with micrococcal nuclease, followed by brief sonication to generate DNA fragments of approximately 200–500 bp. Chromatin was immunoprecipitated with anti-RARα (cat# 62294; Cell Signaling Technology, Danvers, MA, USA) or normal rabbit IgG as a negative control. Immunoprecipitated DNA was purified and analyzed by quantitative PCR using primers spanning the retinoic acid response element (RARE) within the PAI-1 regulatory region. Data were normalized to input and presented as fold enrichment relative to the IgG control.

### 2.12. Real-Time RT-PCR (RT^2^-PCR)

Total RNA extraction, cDNA synthesis and RT^2^-PCR were performed as described before [11]. Primers and corresponding annealing temperatures are listed in Table 1. Relative expression levels were calculated using the 2^−ΔΔCT^ method and normalized to a reference gene, RPS13.

### 2.13. In Vivo Invasion Model

The in vivo invasion model was performed by implantation of tumor cells on the chorioallantoic membrane (CAM) of the chick embryo followed by the analysis of the invasiveness of the implanted cells on the CAM as described previously [14,17]. Briefly, fertilized chicken eggs were incubated in a humidified rotary incubator at 38 °C and 50% humidity for 10 days. At embryonic day 10 (ED10), eggs were candled to visualize major vessels. The chorioallantoic vein was positioned, and a square was marked approximately 1 cm away from the vein’s branching point. A hole was drilled through the blunt end of the egg into the air sac, and a window within the marked square was opened to expose the CAM. GFP-transfected GBM cells (1 × 10^6^) were suspended in 50 μL growth medium with or without tiplaxtinin (Tip, 30 μM) and then implanted onto the CAM. Vehicle (0.3% DMSO) served as control (*n* ≥ 10 per group). The window was sealed, and eggs were incubated for an additional 7 days. At ED17, tumors were excised and gross tumors were imaged using a Nikon ECLIPSE E600 microscope and NIS-Elements Imaging software (v5.20.02; Nikon, Düsseldorf, Germany). Tumor length and width were measured using a digital caliper, and tumor volume was calculated using the formula: volume = (length × width^2^)/2. For histological analyses, samples were fixed in 4% paraformaldehyde, paraffin-embedded, and sectioned at 4 μm thickness for hematoxylin and eosin (H&E) and for immunohistochemistry (IHC) staining of GFP using a specific rabbit anti-GFP antibody (1:250; cat# 66002-1; Proteintech, Wuhan, China). Images were acquired and analyzed using an AxioImager M.2 microscope (Carl Zeiss AG, Oberkochen, Germany) followed by quantitative analysis of tumor cell invasion.

Structurally, the CAM is composed of multiple layers including chorionic epithelium (ChE), mesenchymal layer (M) and allantoic epithelium (AE). To evaluate tumor cell invasion, six fields in each section (20×) with at least two adjacent sections per tumor were randomly captured on IHC-stained sections in the following areas: adjacent to ChE, mid-M, and adjacent to AE. Aggregates of tumor cells with a diameter larger than 50 μm were defined as a cluster. Tumor invasion was quantified using a semi-quantitative scoring system based on invasion depth (*D*) and tumor cluster size (*S*). For each sample, six random microscopic fields were selected for evaluation. The invasion score for each field was calculated as:

Field Score=∑(n×D×S).

The number (*n*) represents the number of tumor clusters. If a single cluster occupied >60% of the field, a ceiling score of 20 was assigned. The total invasion score for each sample was defined as the aggregate sum of the scores from all six fields (Table 2).

### 2.14. Evaluation of the Cytotoxicity of Inhibitors

GBM cells were treated with AGN193109 (2 µM) or tiplaxtinin (30 µM) for 24 h, which is the same condition used for the functional assays. After treatment, cells were harvested, mixed with trypan blue solution (0.4%), and analyzed using a hemocytometer. Cell viability was calculated as the percentage of viable cells (unstained) among the total counted cells (unstained and stained) and was normalized to the corresponding vehicle-treated control groups.

### 2.15. Statistics

All data are presented as mean ± standard deviation (SD) from at least three independent experiments unless otherwise stated. Statistical analyses were performed using GraphPad Prism 9 (GraphPad Software, San Diego, CA, USA). Differences between the two groups were compared using a two-tailed Student’s *t*-test. Comparisons among multiple groups were analyzed by one-way ANOVA followed by Tukey’s post hoc test. A *p*-value < 0.05 was considered statistically significant.

## 3. Results

### 3.1. ALDH1A3 Expression Is Associated with PAI-1 Expression in GBM

**Figure 1:**
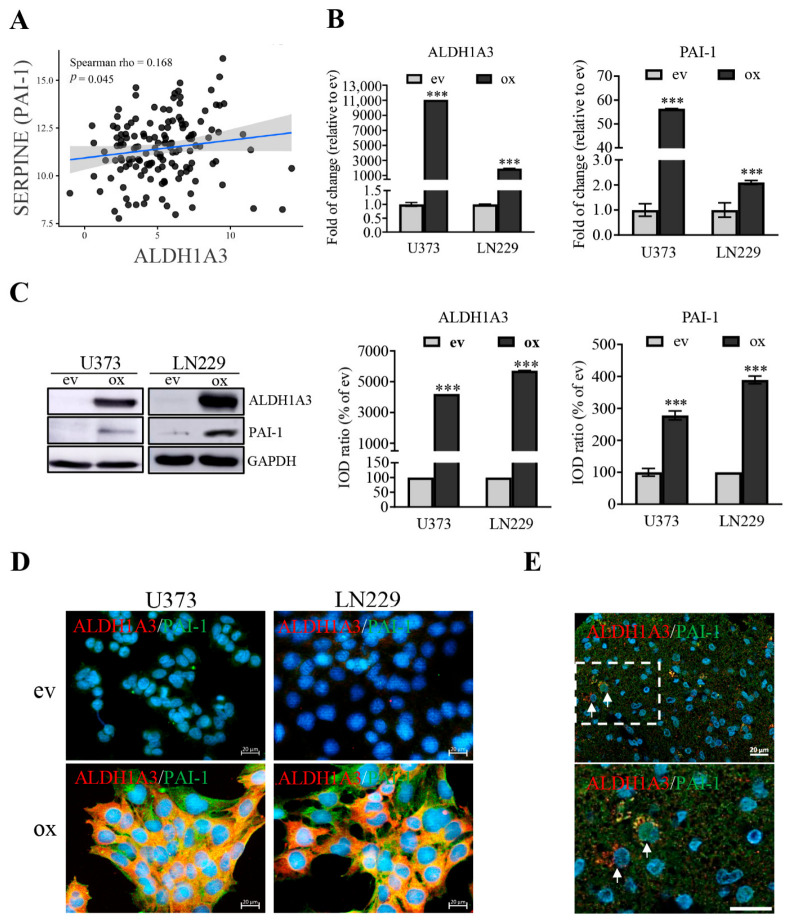
ALDH1A3 expression is associated with PAI-1 in GBM patients and in GBM cells. (**A**): Pearson correlation analysis of TCGA-GBM transcriptomic data showing a positive association between the expression of ALDH1A3 and PAI-1. The cohort was selected according to the histology category in GlioVis (*n* = 153). Cases annotated as IDH-mutant or without available IDH annotation were excluded (*n* = 11), and only IDH-wildtype GBM cases were retained for correlation analysis (*n* = 142). (**B**,**C**): Upregulation of ALDH1A3 and its association with increased expression of PAI-1 were respectively detected by RT^2^-PCR and Western blot analyses in ALDH1A3-overexpression (ox) GBM cells (U373 and LN229) compared with empty vector (ev) transduced cells. IOD, integrated optical density. (**D**): Double immunofluorescence staining of ALDH1A3 (Texas Red) and PAI-1 (FITC) and nuclear staining with DAPI (blue) in ev and ox GBM cells revealed increased ALDH1A3 and PAI-1 immunoreactivity and their cellular co-localization in ox cells. Scale bar, 20 μm. (**E**): Double immunofluorescence staining of ALDH1A3 and PAI-1 in human GBM tumor sections. Representative low-magnification (upper panel) and high-magnification (lower panel) images showed co-localized ALDH1A3 (Texas Red) and PAI-1 (FITC) (arrow) in tumor cells within the infiltrative zone on the GBM patient sections. Scale bar, 20 μm. All experiments were performed in at least three independent replicates. ***, *p* < 0.001, compared with ev.

To assess the relationship between ALDH1A3 and PAI-1 in GBM, we first analyzed the TCGA-GBM cohort using GlioVis and then validated this association in ALDH1A3-overexpressing GBM cells. In the TCGA-GBM dataset, ALDH1A3 expression showed a positive correlation with SERPINE1 (PAI-1) (Spearman r = 0.168, *p* = 0.045; Figure 1A). In TCGA-based survival analysis, patients with high ALDH1A3 expression or high SERPINE1 expression showed shorter overall survival than the corresponding low-expression groups (Supplementary Figure S2). In ALDH1A3-overexpressing cells, RT^2^-PCR detected a robust upregulation of ALDH1A3 mRNA in oxGBM cells accompanied by an increase in PAI-1 by 56.4-fold in U373 cells and 2.1-fold in LN229 cells, respectively (Figure 1B). Western blot analysis confirmed marked elevation of ALDH1A3 and PAI-1 protein expression in oxGBM cells (Figure 1C). Double immunostaining revealed an overlapping cellular expression pattern of ALDH1A3 and PAI-1 in oxU373 and oxLN229 cells, whereas no ALDH1A3 immunoreactivity was detected in ev cells (Figure 1D). Double immunofluorescence staining in human GBM sections revealed overlapping expression of ALDH1A3 and PAI-1 in tumor cells (arrows in Figure 1E). These findings support a positive association between ALDH1A3 and PAI-1 expression in GBM cells.

### 3.2. ALDH1A3 Enhances GBM Migration and Invasion Involving RA/RAR and PAI-1

**Figure 2:**
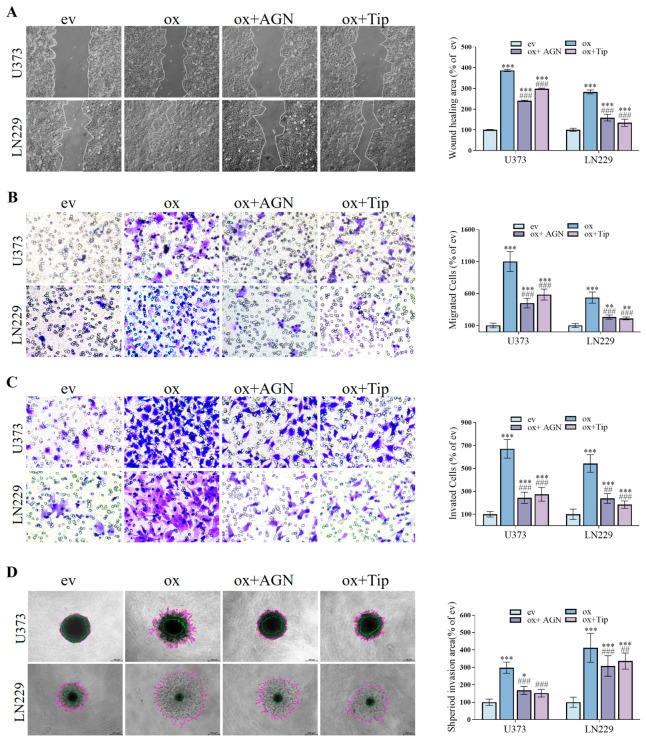
ALDH1A3 overexpression enhanced GBM cell migration and invasion, which was suppressed by inhibition of RA/RAR signaling and PAI-1. Cell mobility was studied in empty vector-transduced (ev) GBM cells and ALDH1A3-overexpressing (ox) GBM cells with or without the treatment of AGN193109 (AGN, 2 μM), or tiplaxtinin (Tip, 30 μM). Control received the treatment with vehicle (0.3% DMSO). (**A**,**B**): Scratch assay and transwell migration assay showed increased migration in oxU373 and oxLN229 cells compared with corresponding ev controls, which was significantly inhibited in the presence of AGN or Tip. (**C**,**D**): Transwell invasion assay and 3D spheroid invasion assay demonstrated an enhanced invasion in oxU373 and oxLN229 cells, and AGN or Tip diminished ALDH1A3 overexpression-mediated cell invasion. All experiments were performed in at least three independent replicates. *, *p* < 0.05; **, *p* < 0.01; ***, *p* < 0.001 compared with ev; ##, *p* < 0.01; ###, *p* < 0.001 compared with ox.

To study the cellular function of ALDH1A3, we performed phenotypic studies using various in vitro models. In the scratch assay, cell migration increased by approximately 3.9-fold in oxU373 cells and 2.8-fold in oxLN229 cells compared with corresponding ev controls (Figure 2A; both *p* < 0.001). ALDH1A3 overexpression-mediated enhancement of cell migration was similarly detected by transwell assay in both cell types (Figure 2B; both *p* < 0.001). We further investigated the invasion behavior by using the transwell invasion assay and the 3D spheroid invasion assay. oxGBM cells showed markedly increased invasive activity in both assays compared with ev controls (Figure 2C,D; *p* < 0.001). These results indicate that ALDH1A3 is a key factor in activating GBM cell mobility.

Given that ALDH1A3 catalyzes RA production and based on the association of ALDH1A3 expression with PAI-1, we next examined whether RA/RAR signaling and PAI-1 were involved in the enhanced GBM cell mobility in ALDH1A3-expressing GBM cells. Interestingly, treatment with the pan-RAR antagonist AGN193109 (AGN) or with a specific PAI-1 inhibitor tiplaxtinin (Tip) significantly reduced the oxU373 cell migration by 38% and 23%, respectively, in the scratch assay (Figure 2A; both *p* < 0.001), and by 59% and 47%, respectively, in the transwell migration assay (Figure 2B; both *p* < 0.001). Both inhibitors also markedly suppressed the invasion activity of oxGBM cells. In oxU373 cells, AGN and Tip treatment reduced transwell invasion by 54% (*p* < 0.001) and 59% (*p* < 0.001), respectively (Figure 2C), and decreased 3D spheroid invasion by 43% and 49% (Figure 2D; both *p* < 0.001), respectively. Similar findings were consistently observed in oxLN229 cells. Cytotoxicity assays confirmed that AGN193109 and tiplaxtinin did not induce significant cytotoxicity under the tested treatment conditions (Supplementary Figure S1).

### 3.3. PAI-1 Knockout Reverses ALDH1A3-Driven Migration and Invasion

**Figure 3:**
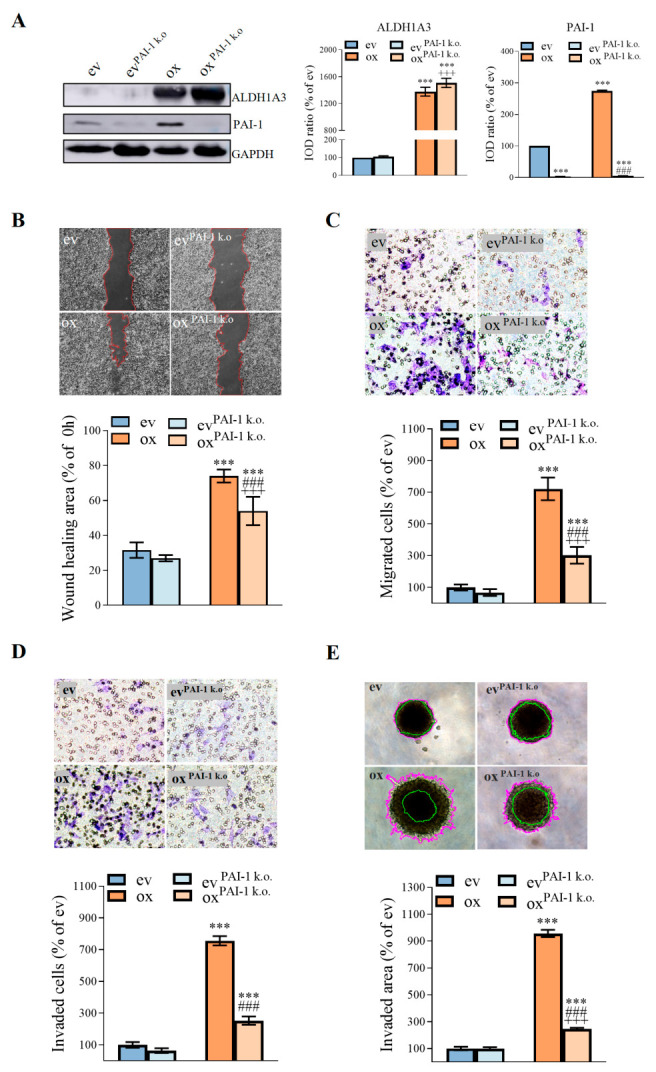
PAI-1 knockout suppresses migration and invasion in ALDH1A3-overexpressing GBM cells. PAI-1 knockout (PAI-1 k.o.) was achieved by CRISPR/Cas9-mediated frameshift technique in empty vector control (ev) and ALDH1A3-overexpressing (ox) U373 cells. (**A**): Knockout efficiency was confirmed by Western blotting. Semiquantification of integrated optical density (IOD) on the blots demonstrated a knockout of PAI-1 in k.o. cells without affecting ALDH1A3 expression. (**B**,**C**): PAI-1 k.o. diminished ALDH1A3-induced migratory capacity as revealed by scratch assay (**B**) and transwell migration assay (**C**). (**D**,**E**): PAI-1 k.o. significantly reduced ALDH1A3-enhanced invasion as shown by the transwell invasion assay (**D**) and by spheroid invasion assay (**E**). All experiments were performed in at least three independent replicates. ***, *p* < 0.001 compared with ev; ###, *p* < 0.001 compared with ox; +++, *p* < 0.001 compared with ev^PAI-1 k.o.^.

To further examine the involvement of PAI-1 in ALDH1A3 signaling and in the mobility activation in oxGBM cells, we generated PAI-1 knockout (PAI-1 k.o.) cells in ev- (evU373^PAI-1 k.o.^) and oxU373 (oxU373^PAI-1 k.o.^) cells using the CRISPR/Cas9 technique by which the translation of PAI-1 protein is disrupted. Genomic validation by Sanger sequencing followed by ICE analysis confirmed efficient editing of the targeted PAI-1 locus. In addition, in silico CRISPOR analysis did not identify high-risk coding off-target sites for the selected guide RNA. Western blotting confirmed the knockout of PAI-1 in both evU373^PAI-1 k.o.^ and oxU373^PAI-1 k.o.^ cells, whereas ALDH1A3 expression remained much higher in oxU373^PAI-1 k.o.^ compared to evU373^PAI-1 k.o.^ cells (Figure 3A; *p* < 0.001). Under these genetic modification conditions, we confirmed the mobility-related phenotypic changes in these cells. As shown in Figure 3, both migration (Figure 3B,C) and invasion (Figure 3D,E) activity of oxU373 cells were markedly inhibited upon PAI-1 knockout.

### 3.4. ALDH1A3 Promotes Tumor Growth and Invasion in the CAM Model Through PAI-1

**Figure 4:**
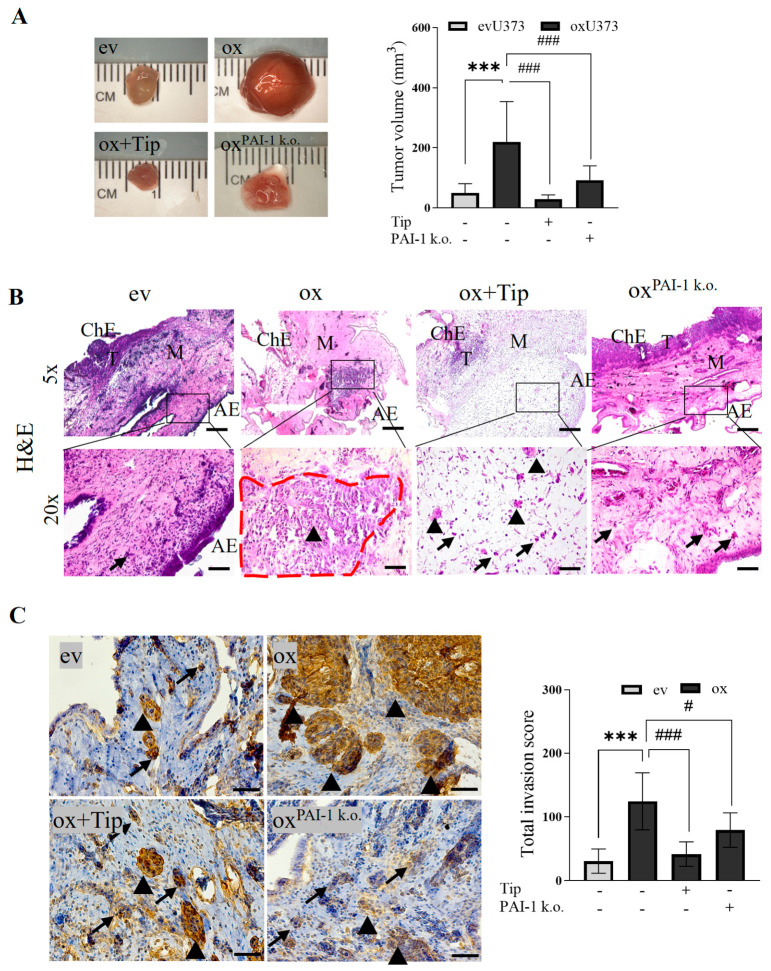
ALDH1A3 overexpression promoted tumor growth and invasion in the CAM model. GFP-transfected U373 cell lines, empty vector control (ev), ALDH1A3-overexpressing cells (ox), and PAI-1 knockout cells (ox ^PAI−1 k.o.^), were implanted onto the CAM of the chicken embryo on ED10. Tiplaxtinin (Tip; 30 μM) or vehicle (DMSO; 0.3%) was added to the cell suspension for the treatment groups on the grafting day. (*n* ≥ 10 per group). (**A**): Representative macroscopic images of excised tumors from different groups and quantification of tumor volume using caliper measurements. Tumors in the oxU373 group were larger than those in the evU373 group. Tip treatment and PAI-1 knockout reduced tumor growth in the oxU373 group. ***, *p* < 0.001 compared with ev; ###, *p* < 0.001 compared with ox. (**B**): Histopathological evaluation of tumor cell invasion in H&E-stained CAM sections. Low-magnification images (5×) show the overall CAM architecture and the distribution of invading tumor cells across the chorionic epithelium (ChE), mesenchymal layer (M), and allantoic epithelium (AE). High-magnification images show the invaded tumor cells (arrows), tumor clusters (arrowheads) and sheet-like tumor invasion areas (red dashed lines). (**C**): Immunohistochemistry staining of GFP. Representative 20× images used for invasion analysis are shown. Arrows and arrowheads indicate tumor cells and tumor clusters, respectively. Semi-quantification indicated a significantly higher invasion score in oxU373 group compared to evU373. Treatment of Tip or PAI-1 k.o. markedly lowered the invasion scores. All groups included more than 10 samples. ***, *p* < 0.001 compared with ev; #, *p* < 0.05; ###, *p* < 0.001 compared with ox.

Next, we performed the chorioallantoic membrane (CAM) invasion model to determine the role of ALDH1A3 in tumor invasion in vivo. GFP-transfected evU373, oxU373, and oxU373^PAI-1 k.o.^ cells were implanted onto the CAM. For the treatment groups, cells were treated with tiplaxtinin (Tip) or vehicle in the cell suspension at the time of grafting (ED10). We observed much larger tumors after 7 days of incubation (ED17) in the oxU373 group compared with the evU373 control (Figure 4A; *p* < 0.001). This increase in tumor burden was significantly reduced by either Tip treatment or PAI-1 k.o. (Figure 4A; both *p* < 0.001).

Histological analysis by H&E staining revealed distinct invasion patterns (Figure 4B). In the evU373 group, the tumor cells were mainly restricted to the implanted site or in the superficial chorionic epithelium layer (ChE layer) (arrows in Figure 4B), whereas in the oxU373 group, tumor cells invaded into the deeper layer, with extensive sheet-like infiltration in the mesenchymal layer (M layer) (Figure 4B, arrowhead and outlined by red dashed lines) and in some cases, invasion extended toward the allantoic epithelium (AE layer) (Figure 4B arrows). This extensive invasion pattern seen in oxU373 cells was reduced by PAI-1 k.o. (Figure 4B).

For a more specific and quantitative analysis of the invaded tumor cells, we performed immunohistochemistry staining of GFP on CAM sections. As shown in Figure 4C, representative 20× GFP-stained images used for invasion scoring revealed a similar distribution pattern of the tumor cells (brown stained cells) in different groups as shown by H&E staining. Arrows indicate invading tumor cells, whereas arrowheads indicate tumor cell clusters. Quantitative analysis demonstrated a significantly higher invasion score in the oxU373 group than in the evU373 group. Of note, the invasion score was clearly lowered by tiplaxtinin treatment (*p* < 0.001) and by PAI-1 knockout (*p* < 0.05) (Figure 4C).

### 3.5. RA Receptor Engagement Promotes PAI-1 Expression

**Figure 5:**
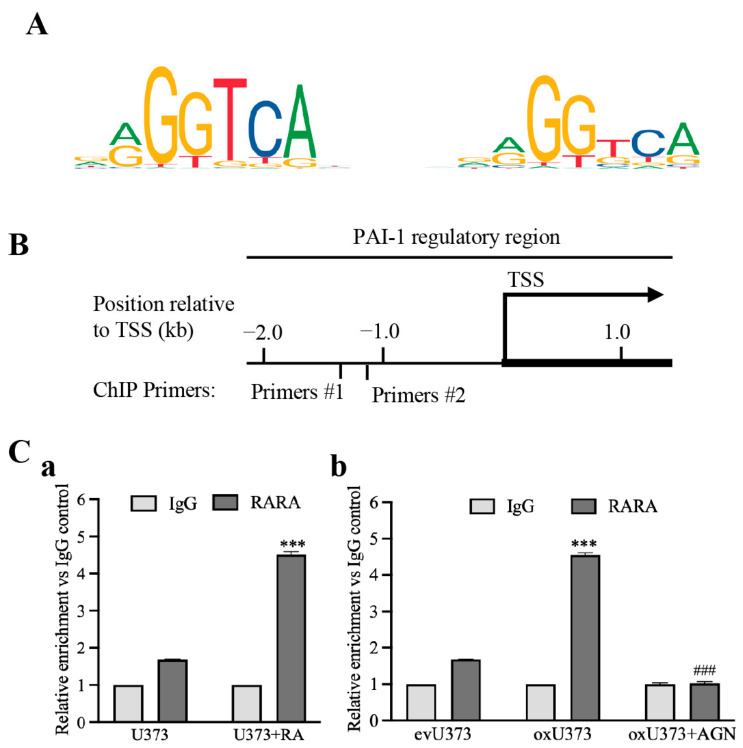
Predicted RAR/RXR binding motif at the PAI-1 locus and RARα occupancy at the PAI-1 regulatory region. Motif analysis and ChIP-qPCR analysis were performed in wtU373 cells treated with vehicle or retinoic acid (RA, 2 μM). In parallel, empty vector-transduced U373 cells (evU373) and ALDH1A3-overexpressing U373 cells (oxU373) were treated with vehicle or with AGN193109 (AGN, 2 μM) as indicated. (**A**): Motif logo of a predicted RARα: RXR binding site (DR5 configuration). The top-scoring site within the analyzed PAI-1 regulatory region (±2 kb relative to the transcription start site [TSS], hg38) is shown (matrix ID: MA1149.1). The height of each letter indicates the relative frequency (information content) of each base at that position, with high conservation observed at the core recognition sites. (**B**): Schematic map of the analyzed PAI-1 regulatory region showing the predicted RAR/RXR binding site and the location of the ChIP-qPCR primer set used for downstream occupancy analysis. (**C**): ChIP-qPCR analysis of RARα occupancy at the indicated PAI-1 regulatory region. Treatment of wtU373 cells with RA increased RARα occupancy at the PAI-1 regulatory region (**a**). Basal RARα occupancy was higher in oxU373 cells than in evU373 controls and was reduced by AGN193109 treatment (**b**). Enrichment is shown as fold enrichment over IgG. IgG served as a negative immunoprecipitation control. All experiments were performed in at least three independent replicates. ***, *p* < 0.001 compared with vehicle-treated wtU373 cells or evU373 controls; ###, *p* < 0.001 compared with oxU373 cells.

Along with our in vitro and in vivo findings and based on the well-known role of ALDH1A3 in RA production, we proposed that ALDH1A3 regulates PAI-1 via RA/RAR. By JASPAR-based motif scanning of the PAI-1 regulatory region, we identified multiple candidate RAR::RXR sites and prioritized a top-scoring RARα: RXRG element for further analysis (Figure 5A). The schematic map illustrates the position of this predicted RAR/RXR binding site relative to the transcription start site (TSS) and the location of the qPCR amplicon used for ChIP-qPCR analysis in the PAI-1 regulatory region (Figure 5B).

**Figure 6:**
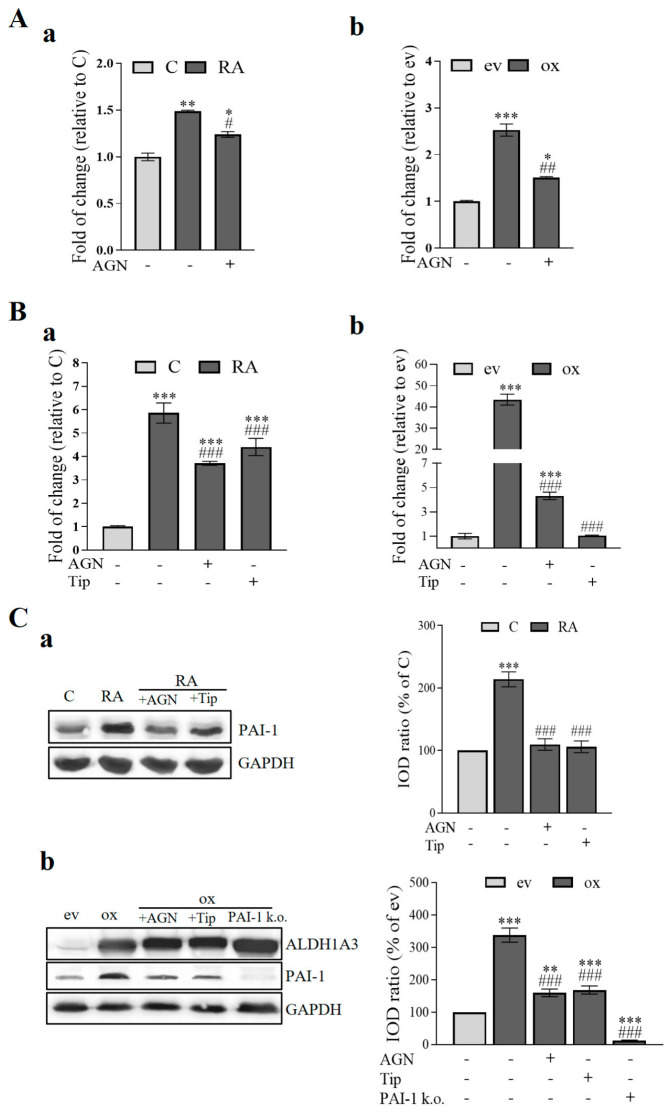
RA and ALDH1A3-associated induction of PAI-1 is reduced by RAR antagonism and PAI-1 blockade. wtU373 cells were treated with RA (2 μM) with or without AGN193109 (AGN, 2 μM). Empty vector-transduced U373 cells (evU373) and ALDH1A3-overexpressing U373 cells (oxU373) were treated with AGN193109 (AGN, 2 μM) or tiplaxtinin (Tip, 30 μM). Control cells were treated with vehicle (DMSO). (**A**): RT^2^-PCR analysis of DHRS3 mRNA expression in wtU373 cells (**a**) and in evU373 and oxU373 cells (**b**). (**B**): RT^2^-PCR analysis of PAI-1 mRNA expression in wtU373 cells (**a**) and in evU373 and oxU373 cells (**b**). (**C**): Western blot analysis and semi-quantification of PAI-1 protein in wtU373 cells (**a**) and in evU373 and oxU373 cells (**b**). IOD, integrated optical density. All experiments were performed in at least three independent replicates. *, *p* < 0.05; **, *p* < 0.01; ***, *p* < 0.001 compared with vehicle control (**C**) or with ev; #, *p* < 0.05; ##, *p* < 0.01; ###, *p* < 0.001 compared with RA-treated or oxU373 cells.

To verify the link between RA/RAR and PAI-1, we first carried out the ChIP-qPCR experiment using wild-type U373 (wtU373) cells. As detected by ChIP-qPCR, treatment of wtU373 with RA resulted in a 2.77-fold increase in RARα occupancy at this site in the PAI-1 regulatory region compared to vehicle control (*p* < 0.001). Consistently, RA treatment significantly upregulated DHRS3, a retinoid-responsive gene used as a transcriptional readout of RA/RAR signaling and was accompanied by increased PAI-1 mRNA expression in wtU373 cells. These effects were clearly inhibited in the presence of AGN193109 (Figure 6B(a)). Western blot analysis confirmed a corresponding increase in PAI-1 protein expression after RA treatment and its reduction following RAR antagonism (Figure 6C).

Using the established ChIP-qPCR method, we further investigated the mechanism by which ALDH1A3 regulates PAI-1. As shown in Figure 5C(b), the basal RARα occupancy at the PAI-1 regulatory region was markedly higher in oxU373 cells than in evU373 controls, and this increase was reduced by treatment with the pan-RAR antagonist AGN193109 (Figure 5C(b); *p* < 0.001), indicating the involvement of RARα in the ALDH1A3-mediated effect. Further experiments detected increased DHRS3 mRNA expression together with PAI-1 mRNA upregulation in oxU373 cells. Upregulation of both genes was suppressed by AGN193109 (Figure 6B(b)). Western blot analysis confirmed these findings at the protein level (Figure 6C(b)). We also examined whether inhibition of endogenous ALDH1A3 affects DHRS3 and PAI-1 expression and cell mobility. For this purpose, T98g cells, which express high endogenous levels of ALDH1A3, were treated with KOTX1, a specific ALDH1A3 inhibitor. We confirmed a significantly higher expression of ALDH1A3 in T98g cells than in the other two GBM cell lines, U373 and LN229 (Supplementary Figure S3A). Inhibition of ALDH1A3 by KOTX1 markedly reduced ALDH1A3 expression as well as DHRS3 and PAI-1 expression (Supplementary Figure S3B). Furthermore, KOTX1 treatment attenuated T98g cell migration and invasion compared with the control (Supplementary Figure S3C,D). These findings strengthened the role of the ALDH1A3-RA/RAR-PAI-1 signaling axis in mediating GBM cell mobility.

## 4. Discussion

ALDH1A3 is a stem cell marker in various types of cancer such as breast cancer, non-small cell lung cancer, melanoma, colorectal cancer, and pancreatic ductal adenocarcinoma [18,19,20,21,22]. In GBM, ALDH1A3 expression has been recognized as a hallmark of the aggressive mesenchymal subtype, which is characterized by increased invasion and poor prognosis [5,23,24]. However, the detailed function and the underlying mechanism of ALDH1A3 in regulating GBM cell migration and invasion remain unclear. Here, we provide in vitro and in vivo evidence for the crucial role of ALDH1A3 in activating GBM cell migration and invasion, which is consistent with our earlier findings in GBM patient sections where ALDH1A3 was dominantly expressed in the infiltration zone of the tumor. By using multiple approaches including genetic overexpression and knockdown as well as by pharmacological interventions, we identified PAI-1 as a key mediator relaying downstream of ALDH1A3-RA/RAR signaling for regulating GBM cell mobility.

ALDH1A3 is a member of the ALDH family and catalyzes the oxidation of retinaldehyde to RA, the final step of RA biosynthesis [23,24]. In mesenchymal glioma stem cells, Li et al. showed that, among ALDH family members, ALDH1A3 is a major catalytic enzyme responsible for RA production and that ALDH1A3 inhibition reduces RA synthesis together with mesenchymal growth-related programs, including clonogenic growth and expression of mesenchymal regulators such as CD44, C/EBPβ, and TAZ [23]. Canonical RA signaling starts with binding to RAR/RXR heterodimers. Upon RA binding, the RAR-RXR heterodimer undergoes ligand-dependent conformational rearrangement that facilitates corepressor release, coactivator recruitment, and transcriptional activation at RA-responsive target loci [25,26]. In this context, RARα serves as an important transcriptional effector of RA signaling, whereas RXR functions as its canonical heterodimeric partner for DNA binding and transcriptional control [25,27]. In the present study, we demonstrated the link between ALDH1A3 and RA/RAR-mediated PAI-1 transcription under various conditions. In wild-type GBM cells with low endogenous ALDH1A3 expression (U373), RA treatment increased the RARα occupancy at the PAI-1 regulatory region, followed by the upregulation of DHRS3, a canonical RA/RAR-responsive readout, and increased PAI-1 expression (Figure 5 and Figure 6); ALDH1A3 overexpression showed effects similar to those observed in RA-treated wild-type cells. The effects observed in both types of cells were diminished by the RAR inhibition. As additional support, in T98g cells, which naturally express high levels of ALDH1A3, the specific ALDH1A3 inhibitor KOTX1 reduced endogenous expression of ALDH1A3 and subsequently downregulated DHRS3 and PAI-1 expression, accompanied by suppressed migration and invasion (Supplementary Figure S3). These findings collectively defined an ALDH1A3-RA/RAR-PAI-1 signaling axis in mediating migration and invasion of GBM cells.

PAI-1 was originally defined as an inhibitor of the uPA/uPAR/plasmin system. Increasing evidence indicates that PAI-1 can promote tumor cell migration and invasion through several complementary mechanisms [28,29]. PAI-1 regulates pericellular proteolysis and extracellular matrix remodeling. It also modulates cell–matrix interactions through vitronectin, uPAR, integrins, and LRP1, thereby affecting adhesion turnover and the repeated detachment steps required for efficient cell motility [30]. In GBM, PAI-1 has been associated with mesenchymal characteristics, poor prognosis, tumor cell dispersal, and orthotopic invasiveness [9]. More recently, GBM migration and invasion were shown to depend on a SMAD2/3-PAI-1 signaling axis [8]. Studies in other tumor types further support a pro-invasive role for PAI-1. In breast cancer, tumor-derived PAI-1 promoted invasion and metastasis by inducing adipocyte-associated collagen remodeling [31]. In lung cancer, PAI-1 has been linked to EMT-associated plasticity, including osimertinib-tolerant states in EGFR-mutated tumors [32]. In esophageal squamous cell carcinoma, PAI-1 promoted migration and invasion through LRP1-dependent AKT/ERK signaling and related stromal crosstalk [29]. TCGA database analysis supported the association between ALDH1A3 and PAI-1 expression and both were associated with shorter overall survival in GBM patients. Experimentally, besides the above-mentioned various models used to establishing the signaling axis and the related functional role in GBM cell mobility, we validated PAI-1 as pivotal mediator in ALDH1A3 signaling by application of a specific PAI-1 inhibitor and specifically by CRISPR/Cas9-mediated PAI-1 knockout in ALDH1A3 overexpression GBM cells in multiple in vitro cell behavior assays (Figure 2 and Figure 3) and in signaling studies (Figure 5 and Figure 6) we well as in vivo CAM model (Figure 4). These studies highlight PAI-1 as an important molecule involved in ALDH1A3-activated signaling and GBM motility. It will be important to further explore how ALDH1A3-associated PAI-1 regulates GBM cell behavior. Our previous findings may shed light on this point. We reported that ALDH1A3 overexpression promoted GBM tumor angiogenesis through paracrine induction of a group of proangiogenic factors including PAI-1 [11]. Thus, ALDH1A3-associated increase in PAI-1 release may stimulate GBM cell activity through microenvironment-mediated mechanisms. Further study is ongoing in our laboratory.

The CAM model is a widely used model to assess tumor growth, invasion, angiogenesis and early drug response in vivo in cancer research including GBM [33,34]. To confirm the role of ALDH1A3 in GBM cell invasion observed in two- and three-dimensional in vitro models, we applied this model by implanting genetically edited GBM cells (GFP-transfected ev-/oxU373 cells and oxU373^PAI-1 k.o.^ cells) and by treatment with specific inhibitors. Based on the microscopically visible CAM structure, we quantitatively analyzed the infiltration depth and extent of the implanted GBM cells. We found that, compared with the evU373 group, oxU373 cells formed significantly larger tumor clusters and displayed a more aggressive invasion pattern characterized by broad sheet-like invasion into deeper CAM layers. By contrast, PAI-1 inhibition or PAI-1 knockout not only reduced tumor growth but also markedly attenuated the extent of tumor cell invasion. These in vivo findings are consistent with those from in vitro models (Figure 2 and Figure 3), supporting a crucial role of ALDH1A3 and its mechanism in GBM cell mobility.

Meanwhile, we are aware of the limitations of the CAM model, as it cannot fully represent the mammalian brain microenvironment and does not reproduce the blood–brain barrier and immune context of orthotopic GBM. Therefore, further validation in patient-derived GBM cell models or orthotopic mouse models will be required in future studies. In addition, PAI-1 promoter-reporter assays under both wild-type and retinoic acid response element (RARE)-mutant conditions should be performed to confirm the functional relevance of the predicted RA-responsive regulatory element.

**Figure 7:**
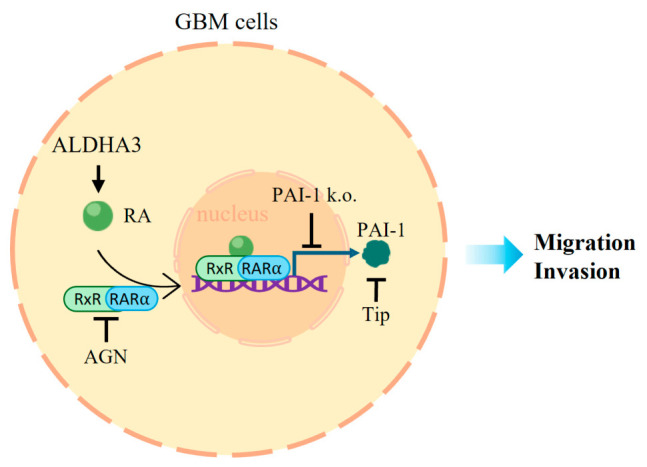
Schematic summary of the signaling axis of ALDH1A3-RA/RAR-PAI-1 that mediates the migration and invasion of GBM cells. As a catalytic enzyme, ALDH1A3 contributes to RA synthesis, which in turn enhances RAR engagement at the PAI-1 regulatory region, thereby inducing PAI-1 transcription. Increased PAI-1 expression contributed to the increased migration and invasion of ALDH1A3-expressing cells. Blockade of the ALDH1A3-RA/RAR-PAI-1 signaling by inhibition of RAR with AGN193109 or by inhibition of PAI-1 with tiplaxtinin, or by PAI-1 knockout suppressed ALDH1A3-mediated tumor invasive phenotype in vitro and in vivo.

We schematically summarize the major findings of the present study as shown in Figure 7. Collectively, our findings support ALDH1A3 as a key driver of GBM cell migration and invasion via a mechanism involving RA/RAR-PAI-1 signaling.

## Figures and Tables

**Table 1:** List of primer sequences and annealing temperatures for RT^2^-PCR.

Primer Name	Sequence	Annealing Temperature (°C)
*ALDH1A3*		60
for	TCTCGACAAAGCCCTGAAGT	
rev	TATTCGGCCAAAGCGTATTC	
*PAI-1*		60
for	GGTTCTGCCCAAGTTCTCCC	
rev	CACCGTGCCACTCTCGTTCA	
*DHRS3*		60
for	TGTATGCAGTAGTCTGGCCG	
rev	CCAGAGAACAATCCTGGCCT	
*RPS13*		60
for	CGAAAGCATCTTGAGAGGAACA	
rev	TCGAGCCAAACGGTGAATC	

for: forward; rev: reverse.

**Table 2:** Histological scoring system for tumor cell invasion on CAM.

Evaluation Metric	Histological Criteria	Score
Invasion Depth (D)	No invasion (restricted on CAM surface)	0
	Invasion into ChE layer	1
	Invasion into M layer	2
	Invasion to the front of the AE layer	3
	Invasion into AE layer	4
Cluster Size (S)	50–100 μm	1
	100–200 μm	2
	>200 μm	3

## Data Availability

The data presented in this study are available in this article (and Supplementary Materials).

## Supplementary Materials

The following supporting information can be downloaded at: https://www.mdpi.com/article/10.3390/cells15121079/s1, Figure S1: Cytotoxicity assay; Figure S2: High ALDH1A3 and PAI-1 expression is associated with overall survival in TCGA-GBM cohort; Figure S3: Inhibition of endogenous ALDH1A3 downregulated the expression of its targets and suppresses GBM cell migration and invasion; Figure S4: Original immunoblots for Figure 1C; Figure S5: Original immunoblots for Figure 3A; Figure S6: Original immunoblots for Figure 6C.
