# Supplementary material for: A Novel Signaling Driven by the Stem Cell Marker ALDH1A3 Promotes Glioblastoma Cell Mobility
Source: Cells. 2026 Jun 14;15(12):1079. doi: 10.3390/cells15121079 (PMC13296712; doi:10.3390/cells15121079)
Supplement: Supplementary file 1 [file cells-15-01079-s001.zip › cells-4295192-supplementary.pdf]

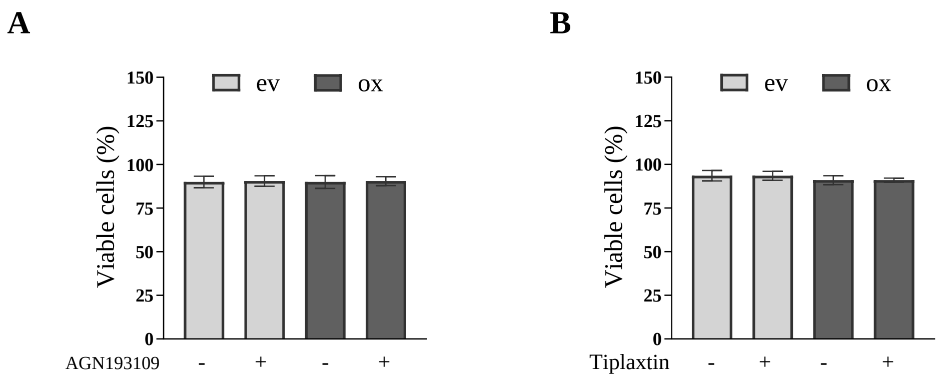

Figure S1. Cytotoxicity assay. evU373 and oxU373 cells were treated with (A): AGN193109 (2 µM) or (B): tiplaxtinin (30 µM). Controls received the corresponding vehicle treatment with DMSO at the same final concentration used for each inhibitor treatment: 0.02% for AGN193109 and 0.30% for tiplaxtinin. Trypan blue staining was performed after 24 h of the incubation. The data did not show significant difference of cell viability among tested groups, suggesting no cytotoxicity under the experimental conditions. Independent experiment was produced at least for three times.

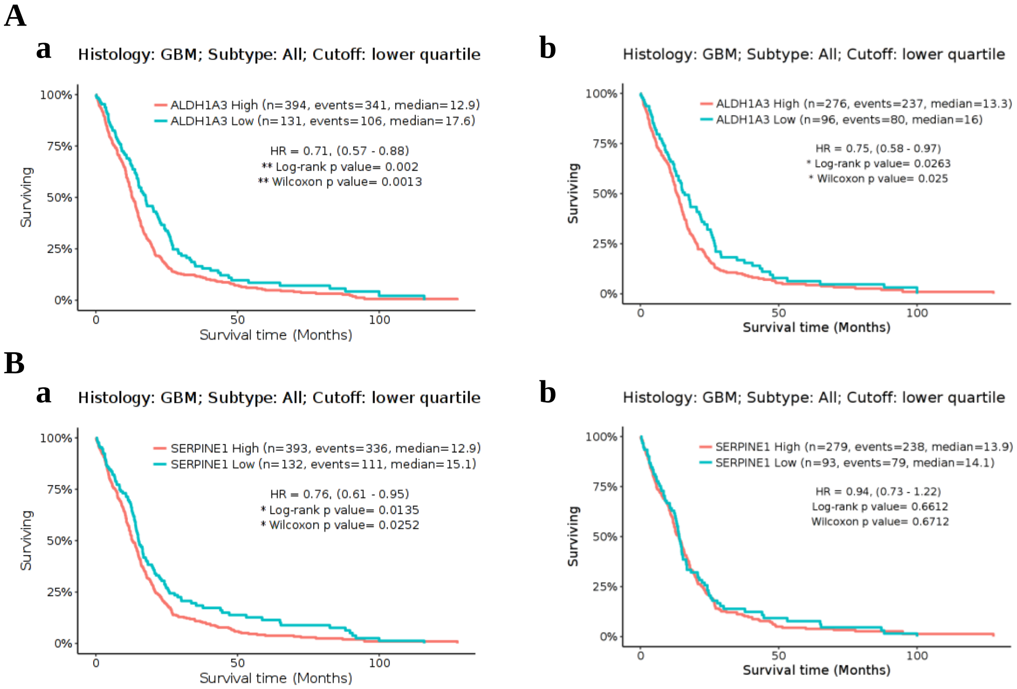

Figure S2. High ALDH1A3 and PAI-1 expression is associated with overall survival in TCGA-GBM cohort.

Kaplan-Meier survival analysis was performed using the TCGA-GBM HG-U133A platform dataset. The curves showed overall survival of GBM patients stratified by ALDH1A3 or SERPINE1/PAI-1 expression. High- and low-expression groups were defined using the lower-quartile expression cut-off in GlioVis. (A-a) Survival analysis according to ALDH1A3 expression in all included GBM cases (n = 525; cut-off value: log2 mRNA expression = 4.21) tiplaxtinin and (A-b) in the IDH-wildtype subgroup (n = 372; cut-off value: log2 mRNA expression = 4.20). (B-a) Survival analysis according to SERPINE1/PAI-1 expression in all included GBM cases (n = 525; cut-off value: log2 mRNA expression = 6.20) and (B-b) in the IDH-wildtype subgroup (n = 372; cut-off value: log2 mRNA expression = 6.30). High ALDH1A3 expression was associated with shorter overall survival in both the full cohort and the IDH-wildtype subgroup, whereas high SERPINE1/PAI-1 expression was associated with shorter overall survival in the full cohort but no statistically significant difference for this association in the IDH-wildtype subgroup. Log-rank p-values, Wilcoxon p-values, hazard ratios (HRs), 95% confidence intervals (CIs), and median survival times are indicated in each panel.

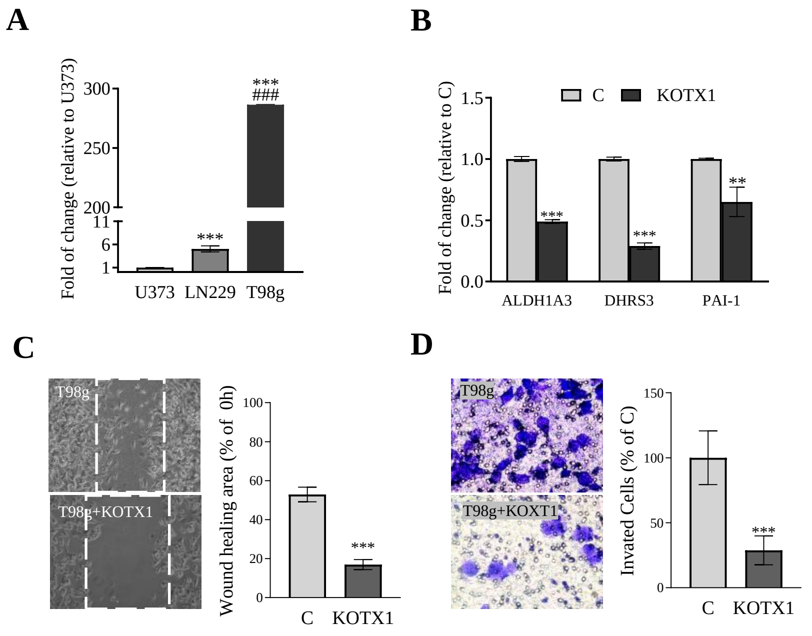

Figure S3. Inhibition of endogenous ALDH1A3 downregulated the expression of its targets and suppressed GBM cell migration and invasion. T98g cells, known expressing high ALDH1A3, were treated with a specific ALDH1A3 inhibitor KOTX1 (1 µM, 24 h). The control (C) received vehicle treatment (DMSO, 0.01%). (A): RT^2-PCR analysis of ALDH1A3 expression in U373, LN229 and T98g demonstrated a significantly higher ALDH1A3 expression in T98g cells compared to U373 and LN229 cells ***, $p < 0.001$ compared with the U373; ### $p< 0.001$, compared to LN229. (B): RT^2-PCR detection of ALDH1A3 RA/RAR specific transcript DHRS3 and PAI-1. The expression of ALDH1A3 was significantly suppressed by the treatment of the inhibitor KOTX1, accompanied by a concomitant downregulation of DHRS3 and PAI-1 expression. These findings indicate that suppression of endogenous ALDH1A3 sufficiently inhibits ALDH1A3-associated RA/RAR-PAI-1 signaling. (C) and (D): Scratch assay (C) and Trans-well invasion assay (D) showed that KOTX1 treatment significantly inhibited the migratory and invasive activity of T98g cells. All experiments were repeated at least for three times. ***, $p < 0.001$ compared with the C.

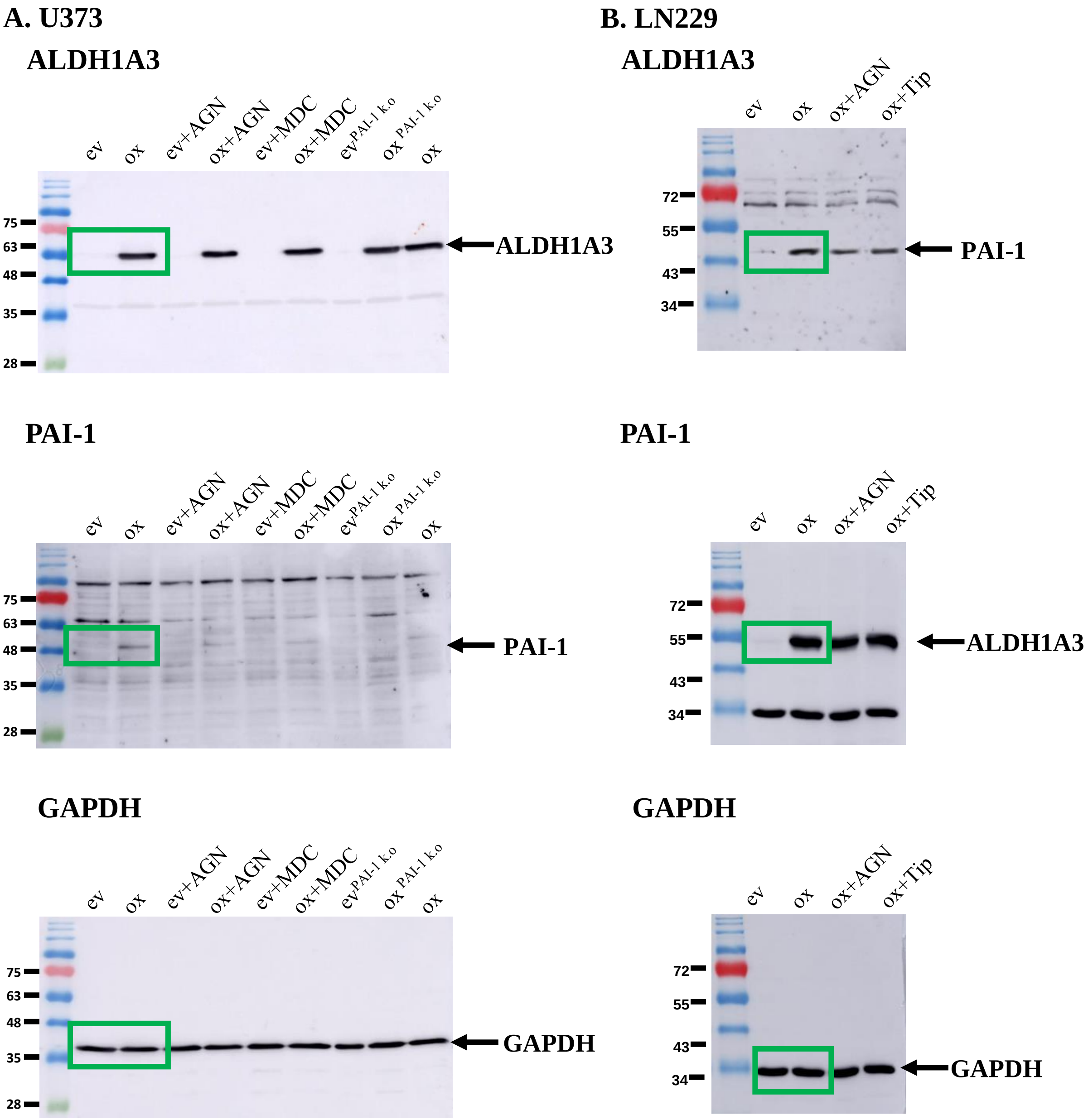

Figure S4. Original immunoblots for Figure 1 C. (A) and (B): ev/oxU373 and ev/oxLN229 cells were collected for western blot. Another subsets were from different treatment group, whose data were not included in Figure 1C. The blots shown in Figure 1C for ALDH1A3, PAI-1 and GAPDH were marked in rectangles.

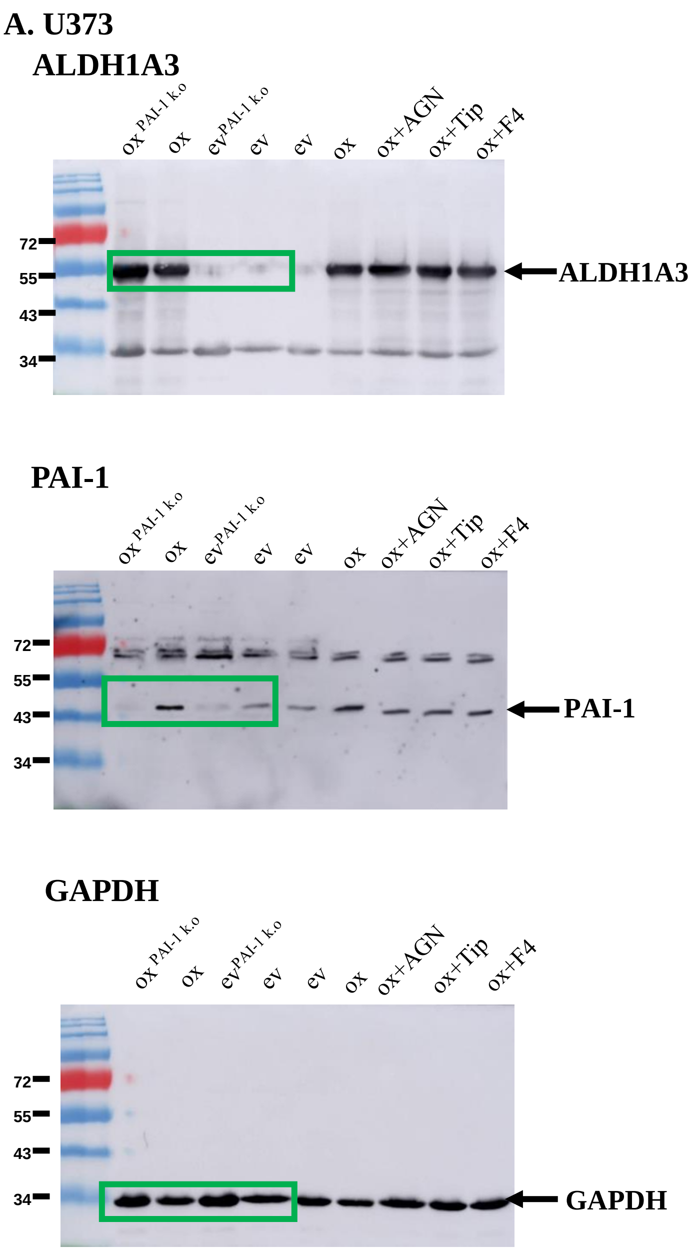

Figure S5. Original immunoblots for Figure 3 A. (A): ev/oxU373 cells were collected for western blot. Another subsets were from different treatment group, whose data were not included in Figure 1A. The blots shown in Figure 3A for ALDH1A3, PAI-1 and GAPDH were marked in rectangles.

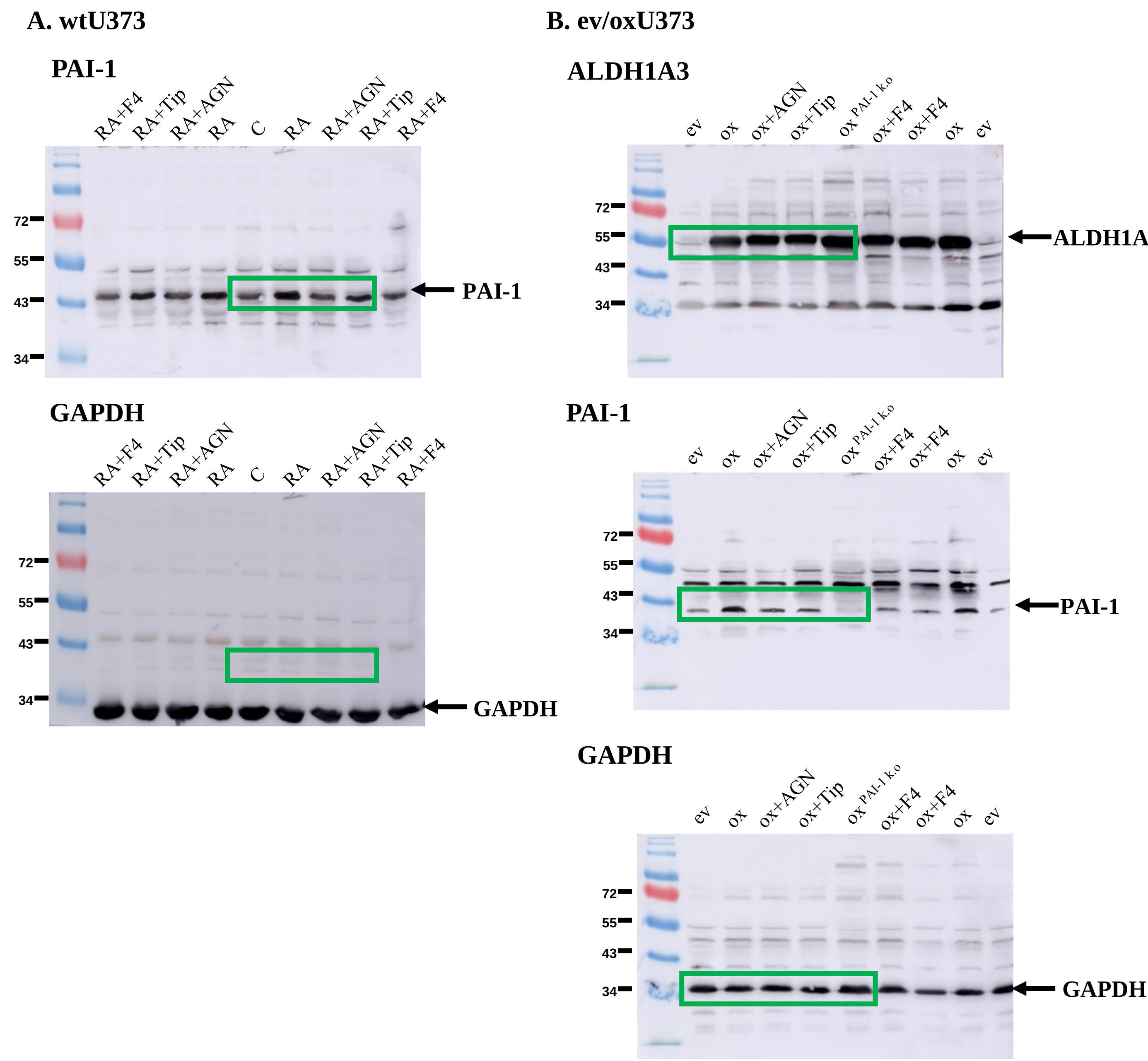

Figure S6. Original immunoblots for Figure 6 C. (A) and (B): wtU373 and ev/oxU373 cells were collected for western blot. Another subsets were from different treatment group, whose data were not included in Figure 6 C. The blots shown in Figure 6 C for ALDH1A3 , PAI-1 and GAPDH were marked in rectangles.
